# Association of alternative healthy eating index and severity of pemphigus vulgaris: A cross-sectional study

**DOI:** 10.1371/journal.pone.0295026

**Published:** 2023-12-11

**Authors:** Maryam Fallah, Anahita Najafi, Kamran Balighi, Maryam Daneshpazhooh, Soraiya Ebrahimpour-Koujan

**Affiliations:** 1 Students’ Scientific Research Center, Tehran University of Medical Sciences, Tehran, Iran; 2 Department of Clinical Nutrition, School of Nutritional Sciences and Dietetics, Tehran University of Medical Sciences, Tehran, Iran; 3 School of Medicine, Tehran University of Medical Sciences, Tehran, Iran; 4 Autoimmune Bullous Diseases Research Center, Tehran University of Medical Sciences, Tehran, Iran; 5 Department of Dermatology, Razi Hospital, Tehran University of Medical Sciences, Tehran, Iran; University of Catania: Universita degli Studi di Catania, ITALY

## Abstract

**Background:**

Evidence on the association between following healthy eating and the severity of pemphigus vulgaris (PV) is scarce. Therefore, the aim of this cross-sectional study aimed to investigate the relationship between adherence to the alternative healthy eating index (AHEI) and the severity of Pemphigus vulgaris disease in adults.

**Methods:**

In this hospital-based cross-sectional study, a total of 138 pemphigus vulgaris cases were studied, of which 108 had pemphigus disease area index (PDAI) ≤15, and 30 had PDAI>15. Dietary intakes were assessed using a valid 168-item food frequency questionnaire (FFQ). To calculate the AHEI, the data received from the diet were used. The subjects of this index received a score of 1–10. The final AHEI was calculated by summing the component scores.

**Results:**

After adjusting for age and sex, we found that individuals with the highest AHEI score were 72% less likely to have increased PV severity compared with those with the lowest score (OR: 0.28; 95% CI: 0.08–0.92, P trend = 0.020). Further control for another potential confounder, intake energy, made the association stronger (OR: 0.24; 95% CI: 0.06–0.88, P trend = 0.017). Also, after controlling the body mass index (BMI) confounding factor, it maintained its strong relationship (OR: 0.24; 95% CI: 0.06–0.88, P trend = 0.016).

**Conclusion:**

The evidence of our study shows that following the healthy eating index is associated with a reduction in the severity of pemphigus vulgaris. Prospective cohort studies are needed to confirm these findings.

## Introduction

Pemphigus vulgaris (PV) is an intraepithelial autoimmune disease with skin acantholysis, flaccid blisters, and erosions of the skin and mucous membranes [1, 2]. The incidence of PV is 6.4/million per year which increases with aging [3]. In Iran, the PV incidence annually is about 1 case per 100,000 people with a 1.6:1 ratio in women than men [4, 5]. PV occurs usually between 40 and 60 years of age [6, 7].

The etiology of PV is still unknown, but its general mechanism is the production of auto-antibodies against intracellular proteins including desmogleins proteins of desmosomes structure [8, 9]. In addition to the genetic factors, most of which are associated with human leukocyte antigen (HLA), affecting the occurrence of PV, many environmental factors can cause the occurrence of this skin disease [10, 11]. Several environmental triggers can be involved in the initiation of auto-immune disease including viral infections, allergens, drugs, and diet [12]. Previously, there was not much attention to the possibilities of environmental contributors in the pathophysiology of PV, however, nowadays increasing evidence has focused on the relation of environmental factors in particular dietary components with PV [12]. Wide ranges of evidence concerning clinical, laboratory, and epidemiological data showed nutritional factors contribute to PV [12, 13]. Due to the mutual mechanisms of the immune system and the digestive system, immunity, and nutrition are closely related and can affect each other [14]. Some nutritional deficiencies may act as a trigger of PV. For example, copper deficiency caused by penicillin can be an initiator of PV [15]. Another study showed that in addition to copper, lower serum levels of zinc and selenium are also related to the occurrence of PV [16, 17]. In the case of nutritional deficiencies, it has been shown that a relation between vitamin C deficiency and initiation of skin diseases like PV [18]. By contrast, intake of some dietary components from sources of fruits and vegetables such as thiols, tannins, isothiocyanates, and phenols from dietary sources are potential inducers of PV [19]. Dietary polyphenols and thiols increase acantholysis through releasing TNF-α and IL-1α from keratinocytes [20, 21]. Other evidence has been proved that tannin contents, can interact with PV medications, and stimulate alveolar macrophages and platelets at stimulation of inflammation [22]. Therefore, understanding the relationship between diet and PV risk is required to focus on the whole dietary pattern rather than single dietary agents. Among dietary indexes that reflect a healthy dietary pattern, the Healthy Eating Index (HEI) is well-known for numerous chronic diseases [23–26]. Based on previous researches, the Alternative Healthy Eating Index (AHEI) which is derived from HEI showed a remarkable association with chronic diseases such as hypertension, cardiovascular diseases, and type 2 diabetes as well [13, 27, 28]. The AHEI-2010 consists of 11 components reflecting different aspects of a healthy diet including vegetables, fruit, whole grains, nuts and legumes, long-chain omega-3 fats (docosahexaenoic acid and eicosapentaenoic acid), polyunsaturated fatty acids, sugar-sweetened drinks and fruit juice, red and processed meat, trans fat, and sodium [29]. The higher adherence to AHEI reduces autoimmune diseases according to nutritional recommendations [30, 31]. So that AHEI can reduce inflammation and amplify the immune system (13). Higher diet quality, which has been measured with AHEI, is associated with lower inflammation [32]. In two studies, it has been shown that the increase in adherence to AHEI-2010 is associated with a decrease in the risk of developing rheumatoid arthritis (RA) in women [28, 33]. Based on our literature review, there is no study on the association of AHEI with PV. However, several studies have reported that dietary components are major contributors to blistering epithermal problems like PV. Finding the relationship between AHEI and PV can open new insight regarding the prevention or control of PV recurrence and move forward the science in this area. Consequently, we can recommend dietary modifications for the control of disease initiation or recurrence. This cross-sectional study was, therefore, designed to investigate the association between AHEI and PV risk among the Iranian population.

## Material and methods

### Study design and participants

We conducted a cross-sectional study on adult population (18–65 years of age at onset of disease) in 2021–2022 (December 2021- May 2022). The diagnosis of PV has been confirmed with immunofluorescent confirmation and serology and histopathology at Razi Hospital (affiliated to Tehran University of Medical Sciences, Tehran, Iran); a referral center for skin disease in Iran. The random sampling of this study is without any gender restriction. The Research Ethics Committee of Tehran University of Medical Sciences in Tehran, Iran approved this study protocol on human subjects (IR.TUMS.MEDICINE.REC.1400.1450). In our study, we described the inclusion criteria related to demographic characteristics (18–65 years of age and male or female), clinical characteristics (diagnosis of PV, stable disease, outpatient, and current or former smoker), and patients with the range of calorie intake is 800–4200. We excluded patients in pregnancy and breastfeeding. Migration, management, and control of pre-existing chronic disease, using nutritional supplementation, adhering to a special diet for weight loss, visiting a nutritionist during the past year, suffering from life-threatened chronic diseases such as cancers, heart failure, liver failure, kidney failure, and other autoimmune diseases, history of inflammatory disorders except for pemphigus vulgaris and malignancy problems. The number of samples, 138 cases, was determined according to types I and II error of 0.05 (N = [(Zα+Z_1-β_)/0.5×ln(1+r/1-r)]^2^ +3. Using r = 0.30, 1-β = 0.95 and α = 0.05) as well as a special formula.

### Assessment of dietary intake

The dietary data were assessed by an approved semi-quantitative food frequency questionnaire (SFFQ), (a gold standard for energy intake, with 168 items completed for all PV participants, which validity and reliability have been proven in the Iranian population, according to usual dietary intakes during last year of study enrollment [34]. The questionnaire was completed by a general practitioner (GP), and approved by a dietitian via face-to-face interview. A food frequency questionnaire (FFQ) consists of a list of beverages and foods to show the usual frequency of consumption over the period queried. The practitioner asks for the usual portion size for every food and beverage. FFQs include questions about quantity consumed due to the standard portion sizes, instead of direct weight. An updated version of Nutritionist IV software, that was adjusted for Iranian foods (version 7.0; N-Squared Computing, Salem, OR, USA), evaluates nutrient intakes and daily energy.

### Assessment of disease severity

The Pemphigus Disease Area Index (PDAI) score evaluates the pemphigus severity by observational and physical examination. PDAI is one of the important scores for measuring the severity of pemphigus, which was introduced by the International Pemphigus Definitions Committee [35]. PDAI can examine many diseases related to skin and mucosal involvement and score the level of involvement [36]. The parameters that make up the cases related to PDAI are mucous membranes, skin, and scalp. The cases related to the skin and scalp are damage and activity [36]. In the mucous membrane parameter, the number of blisters in the body organs is counted [36]. The PDAI score ranges is 0–263: 250 points evaluate the activity of the disease (10 points for scalp, 120 for skin, and 120 points for mucous membranes activity), and 13 points for hyperpigmentation caused by pemphigus inflammation. PDAI is used to measure the extent of the lesion and determine the severity of the disease [37]. The PDAI score cannot examine the mild level of the disease, lesion type, and body surface area (BSA) in patients [38]. After calculating the PDAI score for each patient, we categorized the patients into two groups mild (PDAI less than or equal to 15) and severe (PDAI greater than 15).

### Alternative Healthy Eating Index (AHEI)

We used the Alternative Healthy Eating Index 2010 (AHEI-2010) to evaluate dietary quality. It includes the following items (fruit, vegetables, whole grains, nuts, and legumes, long chain omega-3 fats (docosahexaenoic acid and eicosapentaenoic acid), polyunsaturated fatty acids, sugar-sweetened drinks and fruit juice, red and processed meats, and sodium). Due to a lack of information in the original database, trans fatty acid and alcohol were not counted in this scoring. The highest point limit and the lowest point limit for each item are 0 to 10, so those who had the highest consumption of fruits, vegetables, whole grains, nuts and legumes, long-chain omega-3 fats, and polyunsaturated fatty acids were given a score of 10, and the lowest consumption was given a score of 1. In contrast, for sugar-sweetened drinks and fruit juice, red and processed meat, and sodium intake, the lowest decile was given a score of 10 and the highest decile was given a score of 1. Those in deciles 10, 9, 8, 7, 6, 5, 4, 3, 2 and 1 of these components were given the scores of 1, 2, 3, 4, 5, 6, 7, 8, 9 and 10 respectively. The maximum score of the AHEI-2010 index is 90 and the minimum score is 9. More than 40% of the studied people have a healthy diet according to the score of this index. This index does not assess nutrient intake from using supplements [39].

### Assessment of other variables

The socio-demographic characteristics of the studied community including, age (continues), gender (male/female), marital status (single/married), education (under university/university graduated), residence (urban/rural), duration of disease (continues), PDAI score (continues), be in a high-risk job (yes/no), using supplementation of vitamin and mineral (yes/no), medications (yes/no), rituximab cumulative dose (continues(, a dose of the current use of prednisolone (continues), smoking (smoker/non-smoker), high-risk job (exposing to chemicals/exposing to chemicals), having history of any chronic disease including diabetes, hypertension, cardiovascular disease, hyperlipidemia, cancer, hypothyroidism, non-alcoholic fatty liver disease, polycystic ovary syndrome, viral infections and family history of the mentioned disease were obtained using a general demographic questionnaire. The studied patients were selected from the follow-up clinics, so usually at least 2 months have passed since the initial diagnosis of their disease. The participants of the study have taken initial treatments and were included in the study in monthly visits for follow-up of the disease and maintenance treatments. Due to the chronic nature of PV disease, patients must undergo follow-up treatments until the end of their lives. Based on our criteria for selecting participants, medications and their dosages used by patients, which included immunosuppressive drugs, have been applied between the two groups of PDAI and AHEI. In this study, we measured physical activity with the International Physical Activity Questionnaire (IPAQ), whose validity has been proven in the Iranian population, and expressed it as Metabolic Equivalents-hours per week (MET-h/week) [40]. The IPAQ levels were categorized as mild/moderate/intensive.

The anthropometric indices including height, weight, and waist circumference were measured and BMI was calculated by dividing a person’s weight (in kilograms) by the square of their height. Height was determined using non-stretching tape with 0.5 cm sensitivity without shoes and spacing next to the wall while the shoulders were in a normal position. Weight was calculated without shoes and minimal clothing by using the SECA scale (SECA Scale Corp., Munich, Germany) with 0.5 kg sensitivity. To examine the waist, it was measured from the midpoint between the palpable rib and the iliac crest, a non-stretching tape measure was used according to standard techniques.

### Statistical analyses

We categorized the AHEI score into quartiles before analyses. Categorical variables were presented as numbers and percentages. Also, continuous variables were presented as mean ± SD/SE. The normality of test variables was determined by the Kolmogorov-Smirnov test. Patients were classified according to PDAI and AHEI scores. We used independent t-test for continuous qualitative variables and chi-squared or Fisher’s exact tests for ordinal variables to compare general characteristics and nutritional intakes between two categories of PDAI scores. We used one-way analysis of variance (ANOVA) and chi-square or Fisher’s exact tests to compare these characteristics in the AHEI quartiles. We also investigated the relationship between the total AHEI and its components with the risk of disease severity based on PDAI score components in crude and adjusted models with binary logistic regression test. We considered PDAI by coding (PDAI = 15 = 0, PDAI>15 = 1 as a dummy variable) as the dependent variable (Y) in the regression model. In all multivariate models, Q1 of AHEI was considered as a reference. In crude and adjusted models, we used logistic regression to examine the relationship between total AHEI and the severity of skin and mucous membrane involvement. We considered age (continuous) and sex (male/female) as confounders in the first model. The additional controlling of energy intake (continuous) was done in the second model. BMI was considered in the third model. Finally, in the last model, tobacco use (yes/no), university education (yes/no), corticosteroid use (≤5mg/ 5mg<), and physical activity level (mild/moderate) were added to the co-factors of the previous models. We did a statistical analysis with SPSS software (SPSS Inc, Chicago IL—version 22.0) and a P-value less < 0.05 was considered significant.

## Result

The study participants’ characteristics in the two PDAI groups as well as between the alternative healthy eating index score categories are given in **Table 1.**

**Table 1:** Characteristics and daily dietary intake of the study population by two categories of PDAI score.

Variables	Categories of the Pemphigus patients							
	Across PDAI scores Across AHEI scores							
	PDAI≤15	PDAI>15	P^†^	Q1	Q2	Q3	Q4	P^†^
Number of Patients	N = 108	N = 30		N = 37	N = 32	N = 35	N = 34	
**Demographic Variables**								
Age (year)	48.24±12.44	48.33±14.32	0.972	46.43±13.90	51.53±10.95	46.28±13.78	49.20±11.96	0.280
Sex (Female)	62%	50%	0.235	56.8%	53.1%	62.9%	64.7%	0.753
Weight (kg)	76.31±14.50	76.8±12.22	0.869	73.86±14.30	77.71±13.84	79.15±14.07	75.17±13.67	0.377
WC (cm)	86.94±12.23	85.56±12.46	0.588	83.94±10.91	88.87±13.97	88.45±12.61	85.61±11.30	0.278
BMI (kg/m^2^)	27.98±4.73	27.15±4.44	0.390	26.66±4.16	28.94±5.20	28.59±4.26	27.14±4.82	0.121
Smoking (yes)	11.1%	20%	0.224	13.5%	15.6%	2.9%	20.6	0.164
PAL (Moderate, intensive)	44.4%	46.7%	0.855	40.5%	50.0%	48.6%	41.2%	0.852
University-educated (yes)	29.6%	23.3%	0.498	24.3%	25.0%	22.9%	41.2%	0.289
Marital status (Married)	86.1%	80%	0.400	86.5%	90.6%	71.4%	91.2%	0.077
High-Risk Job (yes)	3.7%	13.3%	0.068	8.1%	3.1%	2.9%	8.8%	0.592
Residence (Urban)	93.5%	96.7%	1.000	100%	96.9%	88.6%	91.2%	0.151
**Medical History**								
Disease Duration (day)	5.37±6.04	2.55±3.71	0.002	3.17±3.73	5.21±6.20	5.35±6.31	5.44±6.29	0.275
PDAI score	3.69±3.85	24.43±11.31	<0.0001	12.70±12.20	8.06±11.63	4.89±6.36	6.82±9.97	0.012
PMH (yes)	57.4%	56.7%	0.942	40.5%	68.8%	62.9%	58.8%	0.092
Supplement Use (yes)	15.7%	10%	0.565	5.4%	9.4%	25.7%	17.6%	0.073
Corticosteroid use (Current dose)	9.79±9.12	28±15.97	<0.0001	16.67±15.84	10.50±9.09	12.97±10.40	14.41±15.67	0.272
RTX use (Cumulative dose)	2.73±2.55	1.53±1.70	0.016	2.60±2.73	2.09±1.79	2.52±2.57	2.63±2.52	0.792
MTX use (yes)	5.6%	0%	0.339	0.0%	6.3%	8.6%	2.9%	0.305
Azathioprine use (yes)	0.9%	6.7%	0.119	5.4%	3.1%	0.0%	0.0%	0.323
Systolic BP (mmHg)	123.77±10.23	125.66±10.96	0.380	123.59±11.13	126.34±9.72	124.42±9.98	122.55±10.64	0.506
Diastolic BP (mmHg)	76.64±6.56	77.16±7.51	0.711	75.21±7.48	78.34±6.77	77.74±6.44	75.94±5.93	0.176

All values are mean ± SD unless indicated

^H^ P values were obtained from independent Student’s t-test, one-way ANOVA, or χ2 test, where appropriate

Patients who are in the higher PDAI group (PDAI>15) statistically have riskier jobs, longer duration of disease, and higher doses of RTX. In terms of the PDAI score and corticosteroid use, patients with higher PDAI had lower doses than the low score group. There was no significant difference between the two groups of PDAI in terms of other demographic characteristics.

We examined the patients in four categories of AHEI score, but there was no significant difference in any of the participant’s factors except the PDAI score in all 4 quartiles.

**Table 2** illustrates the dietary intake of the participants. The dietary consumption of total cholesterol, selenium, and egg were higher among patients with high PDAI score than the low score group. By contrast, the consumption of fiber and vitamin C as well as fruits and vegetables, were lower in the high PDAI group.

**Table 2:** Dietary intakes of study participants across case and control groups as well as across quartile categories of AHEI score*.

Variables	Categories of the Pemphigus patients							
	PDAI≤15	PDAI>15	P^†^	Q1	Q2	Q3	Q4	P^†^
Total energy (kcal/d)	2283.76±642.34	2275.71±672.36	0.952	2055.96±574.15	2200.71±649.99	2276.94±598.61	2609.73±659.59	0.003
**Nutrients**								
Carbohydrates (g/d)	327.09±97.31	323.65±115.20	0.870	289.01±88.99	319.91±103.36	328.60±97.45	370.71±101.37	0.007
Proteins (g/d)	90.01±28.51	91.90±25.88	0.743	81.84±25.82	83.33±25.41	89.63±23.77	107.24±29.80	<0.0001
Fats (g/d)	74.65±28.10	73.32±27.37	0.817	66.91±23.49	70.94±26.11	73.66±30.03	86.42±28.69	0.021
Saturated fats (g/d)	22.21±9.57	22.59±9.96	0.852	20.68±8.12	21.64±9.54	21.45±9.18	25.54±11.18	0.150
Polyunsaturated fats (g/d)	15.48±7.64	14.16±5.97	0.384	12.61±5.00	13.92±6.31	17.09±9.52	17.26±6.81	0.012
Cholesterol (mg/d)	267.00±124.01	334.59±186.15	0.020	281.87±158.36	265.81±145.79	279.75±134.87	298.45±130.23	0.833
Total fiber (g/d)	19.14±6.87	15.61±5.78	0.011	12.94±3.67	16.44±4.57	20.19±6.31	24.21±6.39	<0.0001
Sodium (mg/d)	4814.48±2381.79	4330.00±1995.03	0.311	5346.51±2822.56	1746.31±308.71	2414.50±408.12	1926.19±330.34	0.181
Monounsaturated fatty acids (g/d)	21.79±9.41	21.65±8.48	0.942	20.01±8.22	20.40±8.28	20.70±9.11	26.02±10.07	0.019
Simple sugar (g/d)	46.31±44.85	61.12±79.54	0.187	44.36±34.53	56.51±73.94	46.21±48.59	52.01±57.20	0.789
Vitamin A (μg/d)	1604.76±1015.11	1420.57±1010.89	0.380	1154.06±806.15	1249.10±758.14	1805.41±1135.28	2060.90±1041.98	<0.0001
Vitamin D (mg/d)	1.94±1.43	2.38±1.74	0.156	1.86±1.42	1.63±1.25	1.97±1.59	2.68±1.60	0.027
Vitamin E (mg/d)	4.27±1.77	4.22±1.37	0.889	3.62±1.36	3.83±1.36	4.80±2.25	4.80±1.29	0.002
Vitamin C (mg/d)	178.88±102.39	114.80±61.32	0.001	115.47±58.96	163.94±138.35	178.62±89.26	205.67±74.73	0.001
Vitamin B_6_ (mg/d)	1.58±0.65	1.37±0.55	0.114	1.17±0.4532	1.40±0.5851	1.70±0.67	1.88±0.58	<0.0001
Folate (mg/d)	345.17±113.67	314.96±103.02	0.191	255.28±69.48	306.88±82.43	371.00±102.47	425.77±108.18	<0.0001
Vitamin B_12_ (g/d)	4.83±3.79	5.57±3.42	0.338	4.25±2.47	4.49±5.06	4.49±1.67	6.78±4.40	0.013
Potassium (mg/d)	3727.81±1147.18	3339.45±982.69	0.094	2852.27±817.30	3378.66±863.94	3889.36±1063.43	4500.25±1018.66	<0.0001
Calcium (mg/d)	1060.47±348.82	1074.73±375.86	0.846	937.16±276.68	947.96±251.42	1084.82±343.85	1288.06±413.30	<0.0001
Zinc (mg/d)	9.52±3.21	9.49±3.28	0.957	8.12±2.48	8.57±2.83	9.78±2.81	11.66±3.55	<0.0001
Copper (mg/d)	1.39±0.63	1.24±0.55	0.229	1.02±0.40	1.19±0.62	1.46±0.43	1.78±0.72	<0.0001
Selenium (mg/d)	0.03±0.02	0.05±0.03	0.042	0.02±0.02	0.03±0.01	0.04±0.03	0.05±0.03	0.001
Omega-3 (mg/d)	0.21±0.20	0.24±0.16	0.411	0.13±0.11	0.16±0.10	0.23±0.17	0.34 ±0.27	<0.0001
**Food groups**								
Fruits (g/d)	449.06±259.09	288.13±157.76	0.002	273.78±130.99	373.81±221.09	385.68±210.59	560.29±237.27	<0.0001
Vegetables (g/d)	377.11±180.30	293.76±168.06	0.025	262.57±127.29	304.57±115.95	385.68±210.59	487.67±167.50	<0.0001
White meat (g/d)	60.57±47.49	51.73±30.63	0.337	53.68±35.51	59.54±51.68	57.79±40.51	64.09±50.57	0.805
Red and processed meat (g/d)	41.92±33.32	47.49±57.80	0.500	57.16±55.35	38.93±33.99	31.76±21.54	43.54±35.73	0.048
Fish (g/d)	10.23±14.16	10.13±12.62	0.974	5.38±7.47	7.62±7.91	10.98±10.95	17.11±21.54	0.002
Egg (g/d)	26.37±19.67	40.04±37.76	0.008	29.95±28.67	27.64±28.27	31.32±23.34	28.25±20.55	0.932
Legumes and nuts (g/d)	63.03±42.22	55.27±50.59	0.396	31.50±17.62	55.47±39.95	71.56±30.67	88.84±57.82	<0.0001
Low-fat Dairy (g/d)	317.62±238.35	260.29±242.58	0.389	292.72±190.24	287.29±192.81	312.26±251.59	416.44±293.73	0.087
High-fat Dairy (g/d)	119.67±128.85	90.68±83.30	0.246	104.16±126.74	95.28±109.76	128.59±113.98	124.76±132.33	0.621
Refined grains (g/d)	74.63±78.33	102.89±110.67	0.115	91.91±98.14	86.70±98.46	65.89±66.23	78.39±81.70	0.616
Whole grains (g/d)	53.99±56.78	49.65±57.32	0.712	37.43±44.87	45.99±50.29	52.56±68.92	77.20±54.46	0.022
Salt (g/d)	8.42±5.64	6.94±4.25	0.186	10.07±6.71	8.04±3.96	7.96±5.66	6.14±3.94	0.022
Sugar-sweetened beverages (g/d)	91.00±199.95	59.61±55.00	0.397	97.78±122.55	138.15±331.17	54.34±67.83	49.30±60.08	0.145
Vegetable oil (g/d)	6.26±4.94	4.57±2.81	0.075	6.86±6.41	5.92±4.22	5.52±3.00	5.19±3.92	0.452

All values are mean ± SD.

^†^ANOVA for all variables.

When we analyzed the participants across AHEI score quartiles, we found that participants in the highest category had higher intakes of calories, carbohydrates, total fat, protein, total fiber, monosaturated fats, vitamin A, vitamin D, vitamin E, vitamin C, vitamin B_6_, folate, vitamin B_12_, potassium, calcium, zinc, copper, selenium, omega-3, fruits, vegetables, red and processed meat, fish, legumes and nuts, whole grain in comparison with lowest category. By contrast, salt consumption was lower among participants in the highest quartile of the AHEI score than lowest quartile. There were no significant differences in other dietary variables.

**Table 3** shows the multivariate-adjusted ORs for Pemphigus vulgaris severity in the categories of AHEI. After adjusting for age and sex, people with the highest adherence to AHEI were 72% less likely to develop PV than those with the lowest score (OR: 0.28; 95% CI: 0.09–0.92, P _trend_ = 0.020). Further controlling for a confounding factor, intake energy, strengthened the association (OR: 0.24; 95% CI: 0.06–0.88, P _trend_ = 0.017). Then, after adjustment of the BMI confounder this association remained significant (OR: 0.24; 95% CI: 0.07–0.88, P _trend_ = 0.016). For the last model, we have considered four confounding variables of smoking, university education, corticosteroid use, and physical activity level to other confounding variables, which made the association stronger (OR: 0.18; 95% CI: 0.04–0.82, P _trend_ = 0.012).

**Table 3:** Multivariable‑adjusted ratios for PV severity across different categories of the AHEI.

AHEI quartiles					
	Q1	Q2	Q3	Q4	
	OR	OR 95%(CI)	OR 95%(CI)	OR 95%(CI)	P trend
Crude	1.00	0.38(0.12–1.15)	0.27(0.08–0.87)	0.28(0.09–0.90)	0.017
Model I^†^	1.00	0.36(0.12–1.11)	0.28(0.09–0.89)	0.28(0.09–0.92)	0.020
Model II^‡^	1.00	0.34(0.11–1.07)	0.26(0.08–0.86)	0.24(0.07–0.88)	0.017
Model III^§^	1.00	0.36(0.11–1.14)	0.27(0.08–0.89)	0.24(0.07–0.88)	0.016
Model IV^Ꚑ^	1.00	0.33(0.09–1.19)	0.24(0.07–0.88)	0.18(0.04–0.82)	0.012

	**Disease subtype: having an oral lesion**				
	Q1	Q2	Q3	Q4	*P* trend
Crude	1.00 (Ref)	0.43(0.139–1.319)	0.31(0.10–1.01)	0.23(0.07–0.81)	0.012
Model I	1.00 (Ref)	0.41(0.132–1.301)	0.34(0.10–1.10)	0.23(0.07–0.82)	0.015
Model II	1.00 (Ref)	0.38(0.12–1.23)	0.31(0.09–1.03)	0.19(0.05–0.75)	0.011
Model III	1.00 (Ref)	0.41(0.13–1.32)	0.33(0.10–1.12)	0.18(0.04–0.73)	0.011
Model IV	1.00 (Ref)	0.30(0.08–1.10)	0.26(0.07–0.98)	0.18(0.04–0.87)	0.318

Logistic regression models were used to estimate ORs with 95% CIs.

The Low adherence to dietary diversity is the reference category in the logistic regression model.

The P-trend resulted from a logistic regression test.

^†^Model I: Adjusted for age and sex

^‡^Model II: Further controlled for age, sex, and energy intake

^§^ Model III: Additionally, adjusted for age, sex, energy intake, and BMI

^Ꚑ^Model IV: Adjusted for components in Model III and, smoking, education, corticosteroid use, and physical activity level

When we analyzed the data stratified based on oral involvement (Table 3), we found that individuals in the highest quartile of AHEI-2010 were 77% less likely to have severe disease compared to those in the bottom quartile. This result is unchanged after further adjustment for age and sex. Further adjustment for the energy intake made the association stronger (OR: 0.19; 95% CI: 0.05–0.75, P _trend_ = 0.011). This association remained significant after taking the BMI in the model (OR: 0.18; 95% CI: 0.04–0.73, P _trend_ = 0.011). However, in the last stratified model, there was no significant relationship with the addition of drugs to the previous confounding variables.

## Discussion

This study was conducted to investigate the adherence to alternative healthy eating index 2010 (AHEI-2010) with severity of PV among Iranian PV patients. In this cross-sectional study, we found a significant inverse association between adherence to the AHEI-2010 and the severity of pemphigus vulgaris among the Iranian population, even after adjustment for potential confounders. Based on our stratified analysis by having oral lesions, the highest adherence to AHEI was associated with lower disease severity in the crude and all three adjusted models. To the best of our knowledge, this study is the first study on the association between AHEI-2010 and the severity of pemphigus vulgaris.

Prospective studies have reported several risk factors for the initiation and recurrence of pemphigus vulgaris [10–12, 41–44]. Environmental factors along with genetic ones can also play a role in the development of pemphigus vulgaris [11, 12]. For example, changes in food patterns such as the use of anti-inflammatory diets can improve PV and reduce inflammation in these patients [44]. A diet rich in vegetables, fruits, whole grains, and legumes, as well as limited sweets, fats, salt, and processed products can reduce PV severity [44]. We have concluded that the alternative healthy eating index can reduce the severity of PV; however, no study has yet investigated in this regard. However, various studies have investigated the effect of the AHEI-2010 on various diseases, including cardiovascular diseases, high blood pressure, chronic obstructive pulmonary diseases, and many other chronic diseases [27, 29, 45, 46]. Adhering to AHEI-2010 reduces major chronic diseases such as diabetes and cancer by 16% [23]. Also, the implementation of this index can reduce obesity, prostate cancer, unhealthy obesity, and hip fracture, as well as the risk of death from cardiovascular diseases and cancer [47–51]. In another study, after controlling for confounders, participants with the highest HEI adherence had 66% lower ulcerative colitis than those with the lowest HEI adherence [31]. In a study, the consumption of red meat, margarine, and cheese increases the possibility of ulcerative colitis and Crohn’s disease [52]. However, using more olive oil following the Mediterranean diet does not reduce the incidence of rheumatoid arthritis [53]. By contrast, in another report, higher compliance with HEI was not related to reducing the incidence of arthritis [54]. In a study, cereal fiber consumption did not affect the mortality rate from inflammatory diseases [55]. These controversial findings might be raised from differences in the study design, study population regarding age and sex, sample size, and genetic predisposition relating to PV as well as comorbidities.

p38 mitogen-activated protein kinase (p38 MAPK) signaling can adjust inflammatory responses in autoimmune diseases [56]. However, its inhibitors decrease caspase-3 pro-apoptotic proteinase activity leading to the induction of apoptosis resulting from p38 MAPK activity during acantholysis in PV [56]. Dietary patterns rich in omega-3 fatty acids like AHEI can regulate the transferring of the inflammatory cells to the sites of inflammation and inhibit the production of pro-inflammatory cytokines which can modulate the production of lymphocytes [57]. It also increases the mRNA expression of antioxidant enzymes such as catalase, glutathione peroxidase, and superoxidase dismutase [58, 59]. As a result, it increases the cleaning of the lesion site for tissue regeneration [57, 59]. Moreover, a diet rich in omega-3 increases the levels of GSH-dependent, GSH, and Nrf2-dependent antioxidant defenses and ultimately reduces oxidative stress [60]. The content of DHA and EPA and the ratio of DHA to EPA in the membranes play an important role in the activation of the transcription factor Nrf2 by MAPK-p38 and the MAPK-p38-dependent or independent pathways [60].

Being the first report on Alternative healthy eating index and severity of PV in a most prevalent region and considering several confounding factors into account are strengths of this study. However, some limitations should be considered in the interpretation of results. First, due to the cross-sectional design, causality cannot be inferred. Also, PV patients use various lines of medications which can affect their current dietary intakes. As PV patients were recommended to not intake high-risk food groups including thiols, tannins, phenols, etc. their reports might be not relevant causing recall bias and can lead to some sort of measurement error. Also, we had no relevant data on dietary intakes of alcohol and trans-fat and did not consider it in the construction of the score or the models.

## Conclusion

In conclusion, we found evidence that high adherence to the Alternative Healthy Eating Index 2010 is associated with a reduced likelihood of PV severity progression based on the PDAI among the Iranian population. Although causality cannot be proven in cross-sectional studies, as a finding in our study, there is a possible risk indicator. In the end, we concluded that studies with a larger sample size and perspective design are needed to confirm causality.

## Supporting information

S1 Checklist STROBE statement—checklist of items that should be included in reports of observational studies. (DOCX)
